# Bilateral Intracavernous Carotid Artery Aneurysms Presenting as Diplopia in a Young Patient

**DOI:** 10.1155/2013/209767

**Published:** 2013-03-17

**Authors:** Nikolaos Kopsachilis, Maria Pefkianaki, Gianluca Carifi, Ioannis Lialias

**Affiliations:** ^1^Moorfields Eye Hospital, London EC1V 2PD, UK; ^2^Aristotle University of Thessaloniki, Thessaloniki, Greece

## Abstract

*Introduction*. Bilateral intracavernous carotid artery aneurysms (ICAAs) are extremely rare and difficult to treat. *Case Report*. A 26-year-old female presented in our clinic with acute diplopia due to oculomotor nerve palsy on the left side. Magnetic resonance imaging of the brain showed two heterogeneously enhanced masses indicating bilateral ICAA. An endovascular coil embolization was performed on the left side successfully, resulting in resolution of her symptoms. *Conclusion*. Thorough systemic evaluation in young patients with diplopia can reveal life-threatening underlying pathology and prevent major complications.

## 1. Introduction

Artery aneurysms are found in 5% of the general population and become symptomatic in 9/100.000, most frequently in the fifth and sixth decades of life [1]. Intracavernous carotid artery aneurysms (ICAAs) represent less than 1% of intracranial aneurysms and show a slight female predominance [2]. Bilateral intracavernous carotid artery aneurysms are extremely rare and difficult to treat. 

We report an unusual case of bilateral ICAA in a very young female patient presented as acute diplopia and treated with endovascular coil embolization. 

## 2. Case Report

A 26-year-old woman was referred to our clinic with transient worsening diplopia. She also complained of headaches and had no further general motor or sensory symptoms. The patient had no history of hypertension or hypercholesterolemia and was not on any medications. Visual acuity was 6/5 in the right eye and 6/6 in the left eye. Intraocular pressures were 13 mm Hg both sides. Slit lamp examination was normal, and fundoscopy findings were unremarkable in both eyes. Orthoptic examination showed oculomotor nerve palsy on the left side. Other cranial nerves including abducens and trochlear nerves were intact. No systemic diseases such as Ehlers-Danlos, Paget's disease, or Marfan's syndrome were recorded.

**Figure 1:**
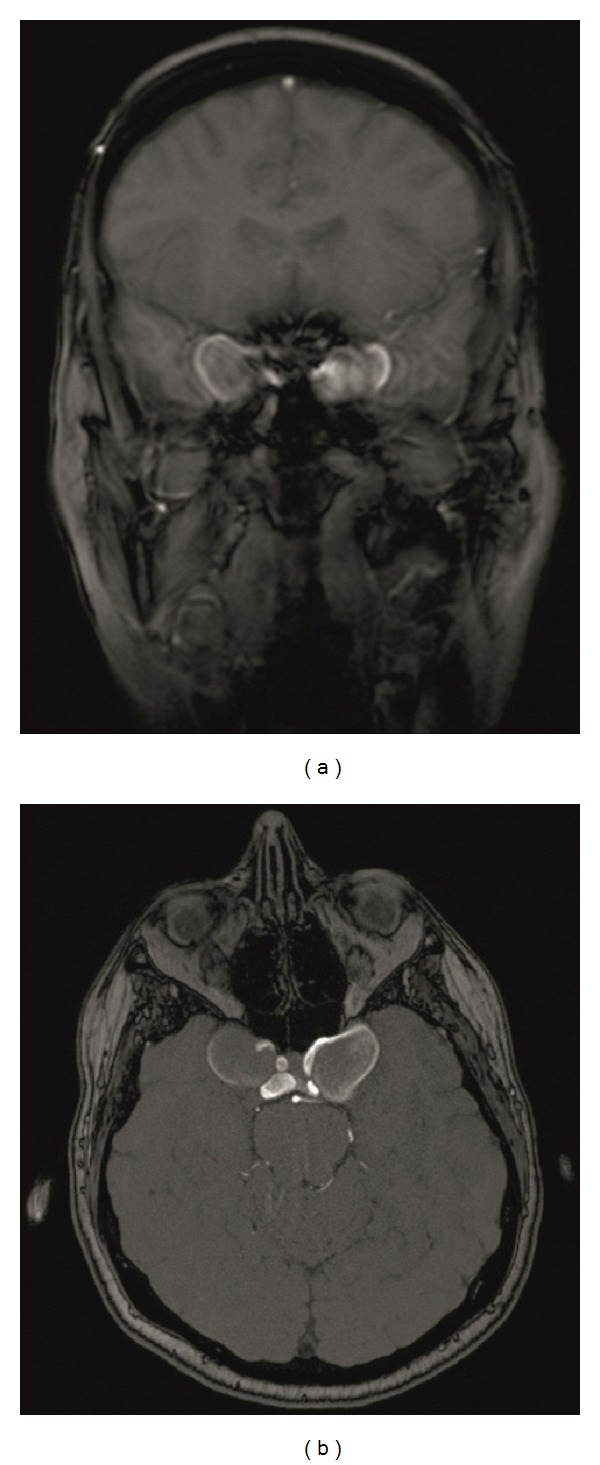
T_1_ weighted MRl of coronal (a) and (b) axial slices in a 26-year-old patient with transient diplopia and oculomotor nerve palsy on the left side. Two heterogeneously enhanced masses and an enhanced thin outer membrane can be recognized, indicating bilateral intracavernous carotid artery aneurysms.

With regard to the clinical findings, magnetic resonance imaging (MRI) of the brain was scheduled. Coronal and axial T_1_ weighted images demonstrated two heterogeneously enhanced masses indicating bilateral ICCA (Figure 1). Bilateral internal carotid arteries were visualized as flow voids encircling the mass.

**Figure 2:**
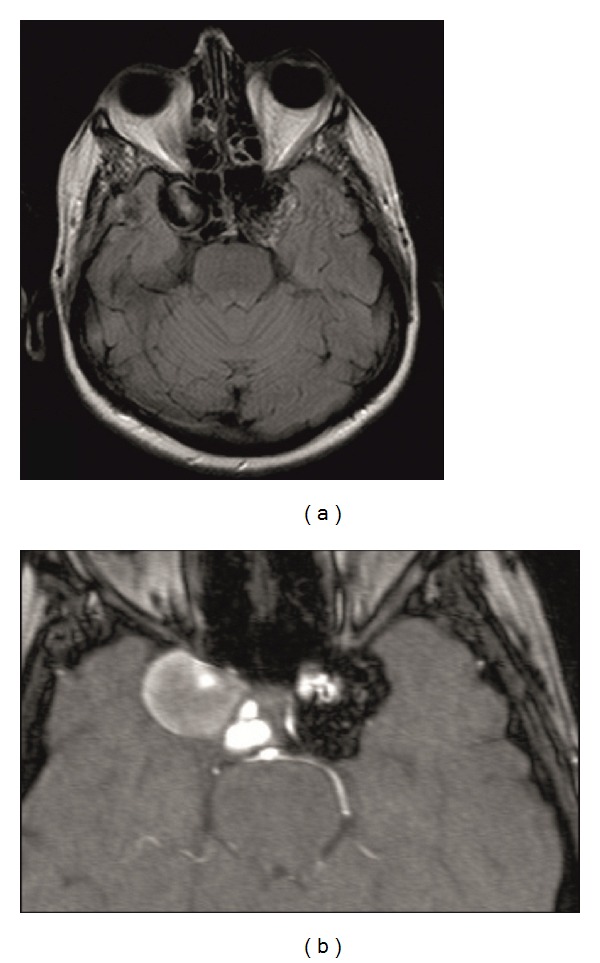
T_1_ weighted MRl axial slices of the same patient 2 days after treatment of the left intracavernous carotid artery aneurysm with endovascular coil embolization (a). Note the slight hypointensity due to thrombosis in the region of the previous left aneurysm (b).

An endovascular coil embolization using a Guglielmi detachable microcoil was performed on the left side uneventfully, in our Department of Neuroradiology. The patient tolerated the procedure well, and there were no major postoperative complications. A postoperative MRI scan showed successful thrombosis of the left aneurysm (Figure 2). One month after embolization, her diplopia had completely resolved. In view of the good postoperative result, an identical procedure on the right side was performed uneventfully 3 months later. At 6 months after initial presentation, the patient had recovered completely and had no recurrence of her diplopia.

## 3. Discussion

The etiology of giant aneurysms is multifactorial and many structural and hemodynamic stress factors have been previously discussed. However, pathogenesis of the cavernous aneurysm is not yet defined, and idiopathic intracavernous aneurysms are the most common [3].

Many bilateral intracavernous aneurysm cases have definite causative factors suggesting weakness of the carotid arterial wall. They can occur after radiation therapy or in association with connective tissue disorders such as fibromuscular dysplasia and Paget's Disease [4]. Furthermore, infectious (mycotic and bacterial) intracavernous aneurysms have been reported in the past [5].

The likelihood of an aneurysm becoming symptomatic is directly related to its size. The most common cause of symptoms (90%) is rupture which can result in severe subarachnoid hemorrhage. Symptoms start with diplopia, photophobia nausea, and vomiting following respiratory arrest with a 50% death ratio. Rupture can also result in arteriovenous fistula or posterior fossa hematoma. In addition, stroke-like symptoms can be created by thrombosis. If the aneurysm is large enough, it compresses surrounding structures and causes progressive neuropathy. In this situation, the symptoms are progressive and less resulting in visual loss, diplopia, ptosis, and other focal deficits. The symptoms are directly related to the cranial nerves (III, IV, VI, VII, and VI) that cross the cavernous sinus [5].

Optimal management of symptomatic giant carotid aneurysms remains controversial. However, present treatment options favor bypass or embolization to direct surgery with very good results, as in our case [6].

To summarize we report an interesting case of bilateral ICAA in 26-year-old female presented as acute diplopia and emphasize the need of a thorough systemic evaluation in young patients with diplopia.
